# The role of TRPV1 in the CD4^+^ T cell-mediated inflammatory response of allergic rhinitis

**DOI:** 10.18632/oncotarget.6653

**Published:** 2015-12-18

**Authors:** Ramachandran Samivel, Dae Woo Kim, Hye Ran Son, Yun-Hee Rhee, Eun Hee Kim, Ji Hye Kim, Jun-Sang Bae, Young-Jun Chung, Phil-Sang Chung, Eyal Raz, Ji-Hun Mo

**Affiliations:** ^1^ Department of Otorhinolaryngology, Dankook University College of Medicine, Cheonan, South Korea; ^2^ Beckman Laser Institute Korea, Dankook University College of Medicine, Cheonan, South Korea; ^3^ Department of Otorhinolaryngology-Head and Neck Surgery, Boramae Medical Center, Seoul National University College of Medicine, Seoul, South Korea; ^4^ Department of Premedical Course, Dankook University College of Medicine, Cheonan, South Korea; ^5^ Department of Medicine, University of California, San Diego, La Jolla, California, USA; ^6^ Clinical Mucosal Immunology Study Group

**Keywords:** allergic rhinitis, BCTC, CD4 T lymphocyte, OVA, TRPV1, Immunology and Microbiology Section, Immune response, Immunity

## Abstract

Transient receptor potential vanilloid 1 (TRPV1), which has been identified as a molecular target for the activation of sensory neurons by various painful stimuli, was reported to regulate the signaling and activation of CD4^+^ T cells. However, the role of TRPV1 in CD4^+^ T cell in allergic rhinitis remains poorly understood. In this study, TRPV1 expression was localized in CD4^+^ T cells. Both knockout and chemical inhibition of TRPV1 suppressed Th2/Th17 cytokine production in CD4 T cells and Jurkat T cells, respectively, and can suppress T cell receptor signaling pathways including NF-κB, MAP kinase, and NFAT. In TRPV1 knockout allergic rhinitis (AR) mice, eosinophil infiltration, Th2/Th17 cytokines in the nasal mucosa, and total and ova-specific IgE levels in serum decreased, compared with wild-type AR mice. The TRPV1 antagonists, BCTC or theobromine, showed similar inhibitory immunologic effects on AR mice models. In addition, the number of TRPV1^+^/CD4^+^ inflammatory cells increased in the nasal mucosa of patients with AR, compared with that of control subjects. Thus, TRPV1 activation on CD4^+^ T cells is involved in T cell receptor signaling, and it could be a novel therapeutic target in AR.

## INTRODUCTION

Transient receptor potential vanilloid 1 (TRPV1) is a polymodal ionic transducer stimulated by a broad range of thermal, mechanical, and chemical stimuli [1]. TRPV1 is expressed *in vivo* in a multitude of non-neuronal cell types in the kidney, pancreas, testes, uterus, spleen, stomach, small intestine and liver mucous gland as well as in neuronal cells [2, 3]. Recently, TRPV1 expression was also confirmed in the airway epithelium [4]. Expression of TRPV1 is markedly up-regulated in the bronchial epithelia and nasal mucosa of patients with refractory asthma [5-7]. Moreover, activation of TRPV1 induces thymic stromal lymphopoietin (TSLP) release in the airway epithelia, which has been linked to allergic airway inflammation in humans [8]. Despite the fact that TRPV1 was up-regulated in rhinitis, there has been little translation to clinical efficacy. In double-blinded randomized cross-over studies, the topical intranasal TRPV1 antagonist, SB-705498 did not alleviate allergen-induced or cold dry air-elicited symptoms in allergic or non-allergic rhinitis [9-13]. This mismatch between the over-expression of TRPV1 in nasal mucosa and the lack of clinical efficacy of TRPV1 antagonist treatment draws our attention to a possible alternative target cell type, immune cells. Interestingly, in a recent study, TRPV1 was found to be functionally expressed in CD4^+^

T cells and to contribute to T cell receptor (TCR)-induced Ca^2+^ channel influx, TCR signaling, and T cell activation [14]. Until now, the expression and functional role of TRPV1 in immune cells including CD4^+^ T cells had not been investigated in allergic rhinitis (AR).

Thus, this study was designed to assess whether TRPV1 activates TCR signaling during the allergic airway inflammatory response, using *in vivo* and *in vitro* models.

## RESULTS

### Expression of TRPV1 in CD4^+^ T lymphocytes

**Figure 1:**
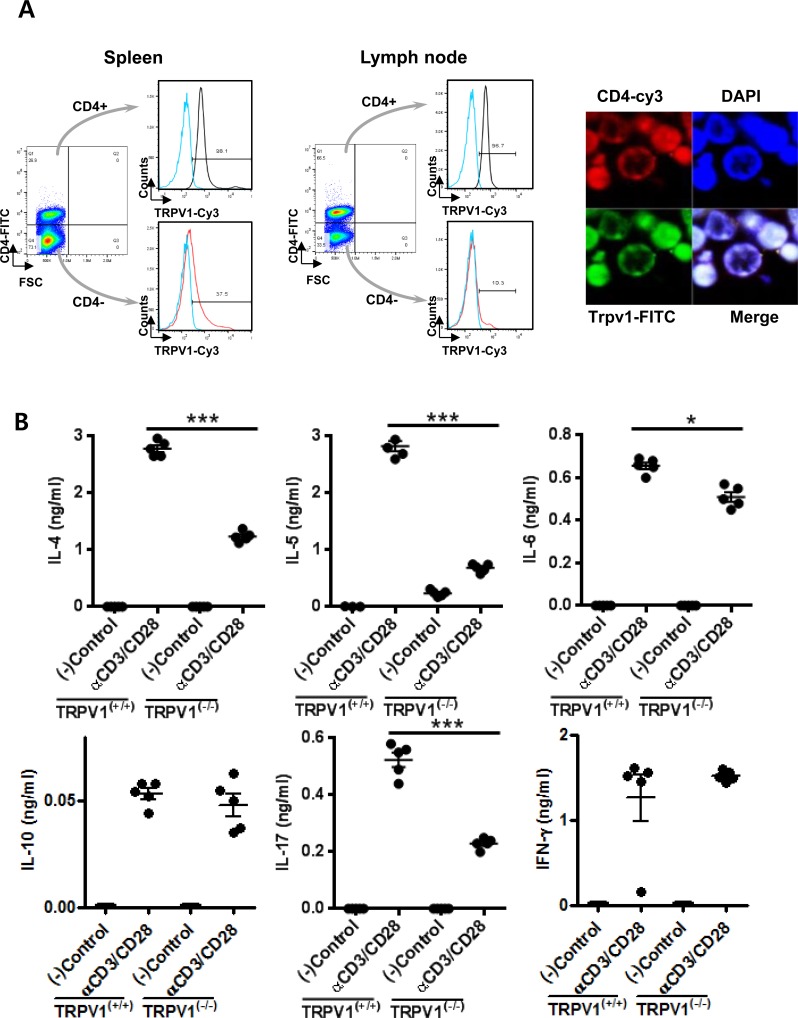

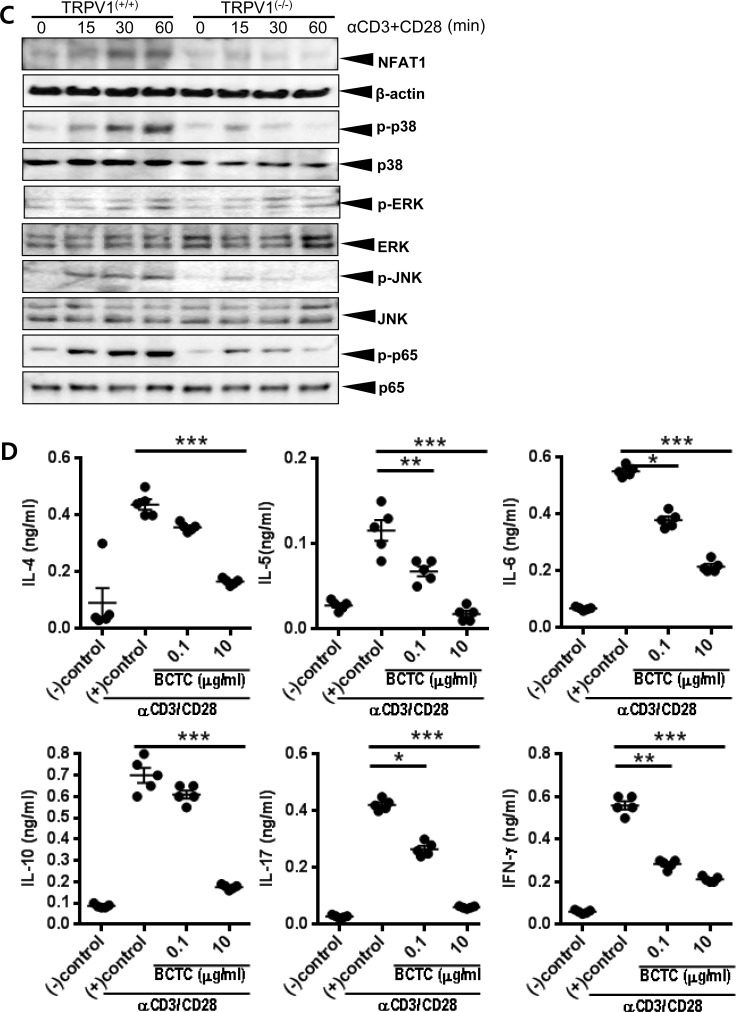

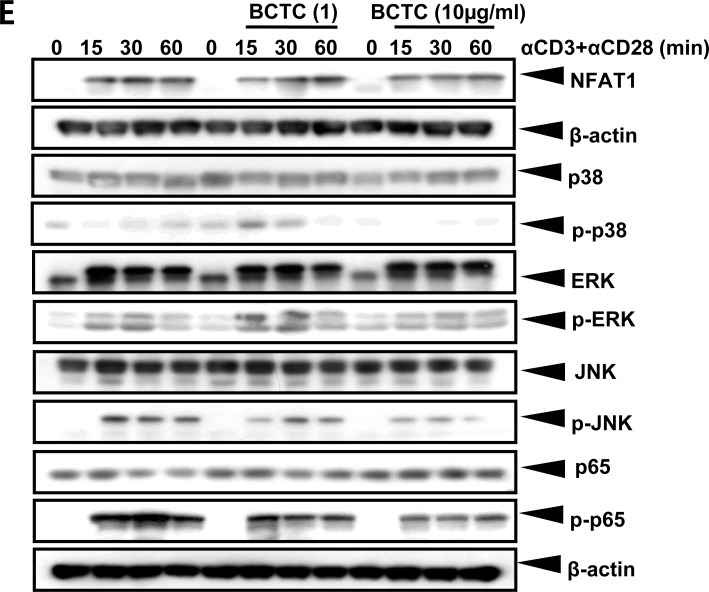
Expression of TRPV1 and its regulation of cytokine production and T cell receptor signaling in CD4^+^ T lymphocytes **A.** The expression of TRPV1 protein in the CD4^+^ T cells was analyzed by FACS and confocal microscopic analyses. **B.** Protein concentrations of the cytokines IL-4, IL-5, IL-6, IL-10, IL-17, and IFN-γ secreted by CD4^+^ T cells isolated from TRPV1^(+/+)^ and TRPV1^(−/−)^ mice splenocytes after stimulation with αCD3/CD28. **C.** The immunoblotting results of T cell recptor signaling pathways in CD4^+^ T cells isolated from TRPV1^(+/+)^ and TRPV1^(−/−)^ mice. Phospho-p65 represents NF-κB activity and phospho-p38, phospho-ERK and phospho-JNK represents MAPK pathway. **D.** The secreted protein concentrations of the cytokines IL-4, IL-5, IL-6, IL-10, IL-17, and IFN-γ in the culture media of human Jurkat T cells pre-incubated with BCTC after stimulation with αCD3/CD28 were determined by ELISA. **E.** T cell receptor signaling pathways in human Jurkat T cells with or without preincubation of TRPV1 inhibitor, BCTC. TCR was activated by anti-CD3/CD28 and two different concentration of BCTC was used. The statistical *P* values are presented as * (*P* < 0.05), ** (*P* < 0.01), and *** (*P* < 0.001).

Since CD4^+^ T cells play a central role in the adaptive immune response, we first assessed whether CD4^+^ T cells from mice expressed TRPV1. We first analyzed the expression of TRPV1 in CD4^+^ T cells from spleen and lymph nodes, using FACS and confocal microscopic analyses. In spleen, 98.1% of CD4^+^ T cells and 37.5% of CD4^+^ cells expressed TRPV1 and in lymph node, 96.7% of CD4^+^ T cells and 10.3% of CD4^−^ cells expressed TRPV1. The results showed that TRPV1 was highly expressed in CD4^+^ T cells. Additionally, confocal microscopy for TRPV1 and CD4 protein expression using fluorescein isothiocyanate-conjugated TRPV1 and Cy3-conjugated CD4 antibodies showed that TRPV1 protein was co-localized with CD4 in CD4^+^ T cells at high levels (Figure 1A).

### TRPV1-mediated TCR signaling in CD4^+^ T cells

CD4^+^ T cells were isolated from the spleens of TRPV1^(+/+)^ and TRPV1^(−/−)^ mice and stimulated with αCD3/CD28, and then their secreted cytokine profiles and TCR signaling pathway activities were assessed. Stimulated CD4^+^ T cells from TRPV1^(−/−)^ mice secreted significantly lower levels of the cytokines (IL-4, IL-5, IL-6, and IL-17), compared with those of the stimulated CD4^+^ T cells from the TRPV1^(+/+)^ mice (Figure 1B). However, IL-10 and IFN-γ levels were not significantly decreased in TRPV1 knockout splenocytes. CD4^+^ T cells from the TRPV1^(+/+)^ mice exhibited increased phosphorylation of p38, ERK, JNK, and NF-κB p65, and also showed increased NFAT1 activation at 15, 30, and 60 min after αCD3/CD28 stimulation (Figure 1C); however, these responses to stimulation were down-regulated significantly in the stimulated CD4^+^ T cells from TRPV1^(−/−)^ mice. These findings suggest that TRPV1 is required for the proper transduction of TCR signaling.

Furthermore, we investigated the effect of TRPV inhibition on cytokine expression and TCR signaling using human Jurkat T cell lines pre-incubated with different concentrations of a TRPV1 inhibitor, BCTC [*N*-(4-Tertiarybutylphenyl)-4-(3-cholorphyridin-2-yl tetrahydropyrazine -1(*2H*)-carboxamide; 0.1, 1, and 10 μg/ml]. Secretion of the cytokines IL-4, IL-5, IL-6, IL-10, IL-17, and IFN-γ by Jurkat T cells significantly decreased following BCTC treatment in a concentration-dependent manner compared with that of the untreated control (Figure 1D), which is concordant with the similar results obtained from TRPV1^(−/−)^ CD4^+^ T cells. To examine whether BCTC inhibits the activation of TCR signaling, the signal transduction pathway was analyzed in αCD3/CD28-stimulated Jurkat T cells. BCTC pretreatment of the stimulated Jurkat T cells led to a significant down-regulation of the phosphorylation of p38, JNK, ERK, and p65, and also reduced the activation of NFAT1 at different time points, compared with those of the untreated Jurkat T cells (Figure 1E), which is also consistent with the results obtained from TRPV1^(−/−)^ CD4^+^ T cells. Hence, these results show that the suppression of TRPV1 inhibits TCR-mediated signaling pathways including NF-κB, NFAT, and MAPK in CD4^+^ T cells.

### TRPV1 knockout reduces inflammatory responses in OVA-sensitized mice

**Figure 2:**
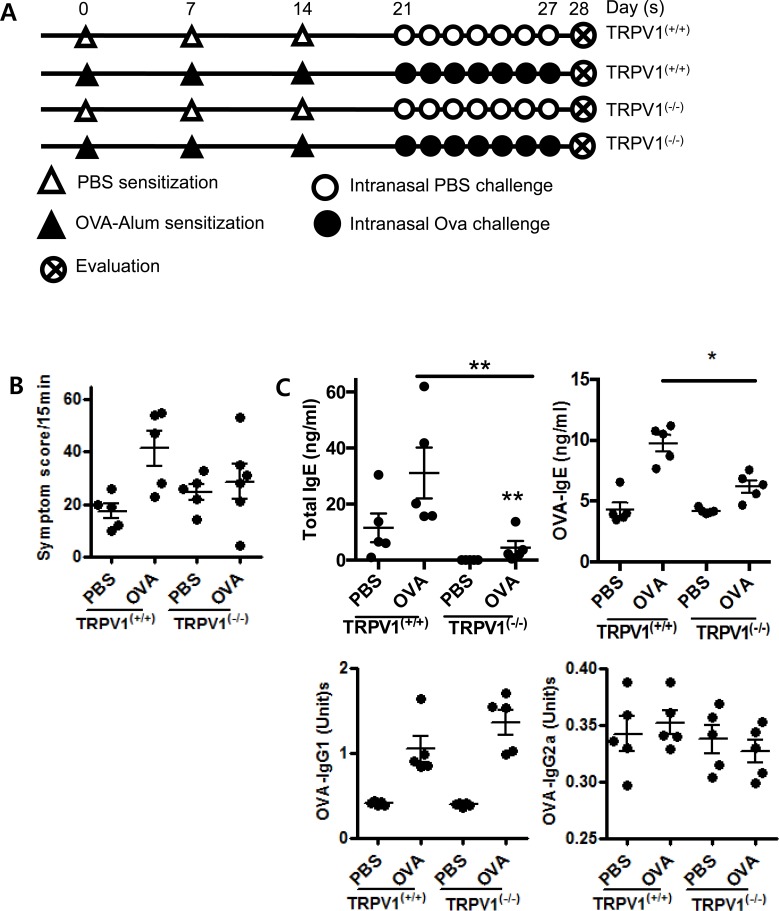

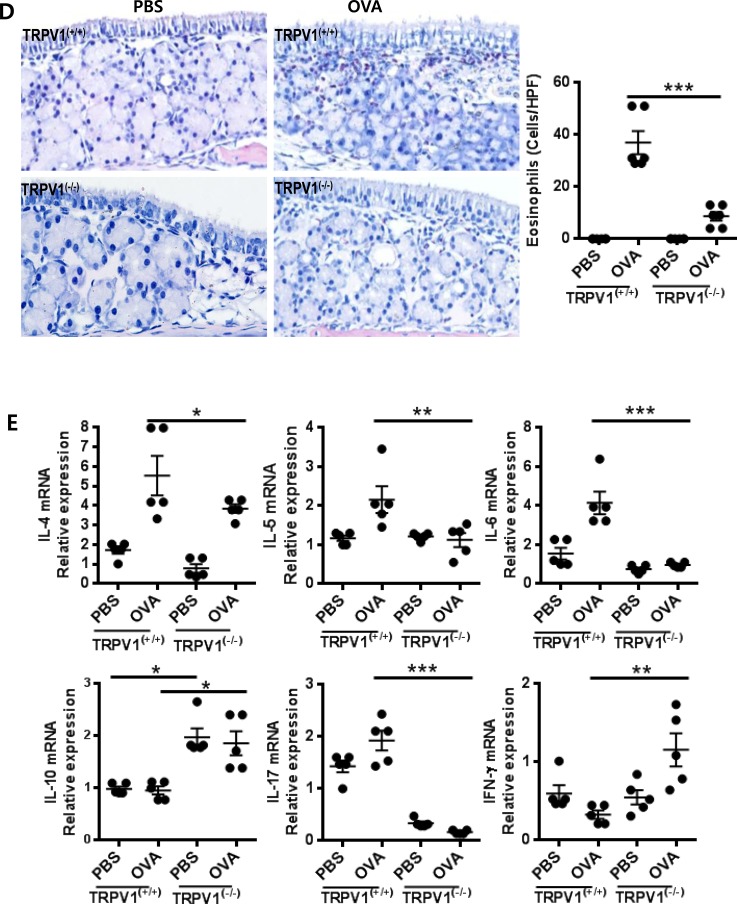

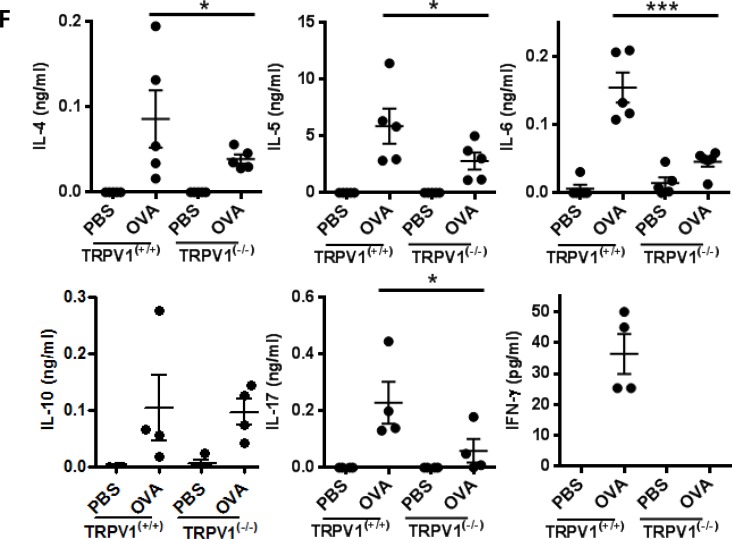
TRPV1 regulates inflammatory responses in OVA-sensitized mice **A.** The experimental protocol for the establishment of the allergic rhinitis model. TPRV1 knockout and wild type mice were used. **B.** Symptoms score of nasal rubbing and sneezing for 15 minutes after ovalbumin challenge **C.** The total OVA-specific IgE, IgG1, and IgG2a levels in the serum, as determined by ELISA, in the treated mice. **D.** The histopathological changes in the nasal mucosal septa of TRPV1^(+/+)^ and TRPV1^(−/−)^ mice were quantified based on the infiltration of eosinophils, as determined by Sirius-red staining (400× magnification). **E.** Quantitative real-time PCR results using the Taqman^®^ probe method showing the expression of cytokines in the nasal mucosa. **F.** Protein concentrations of cytokines, as determined by ELISA, in the supernatants of splenocytes from the treated mice. The spleen single-cell suspensions were cultured (3 × 10^6^ cells/ml) with or without OVA (1 mg/ml), and the culture supernatants were collected after 72 h. The statistical *P* values are presented as * (*P* < 0.05), ** (*P* < 0.01), and *** (*P* < 0.001).

T cells are one of the most important players in allergic inflammation, so we aimed to evaluate whether the suppression of TRPV1 could influence allergic inflammation using a mouse model of allergic rhinitis. To investigate the role of TRPV1 in the allergic immune response, we compared TRPV1^(−/−)^ and TRPV1^(+/+)^ mice using the OVA-sensitized allergic rhinitis model. In both types of mice, TRPV1 expression was confirmed by PCR (Supplementary Figure 1) and further experiments were conducted using the experimental schedule (Figure 2A). The OVA-challenged TRPV1^(−/−)^ mice showed decreased nasal rubbing and sneezing scores compared with those of the OVA-challenged TRPV1^(+/+)^ mice (Figure 2B). Total and OVA-specific IgE levels were significantly decreased in the OVA-challenged TRPV1^(−/−)^ mice compared with those of the OVA-challenged TRPV1^(+/+)^ mice (Figure 2C), and Sirius red staining showed that nasal eosinophil infiltration was also decreased in the OVA-challenged TRPV1^(−/−)^ mice (Figure 2D).

Nasal mucosal cytokine mRNA profiles (IL-4, IL-5, IL-6, IL-10, and IL-17) were investigated by RT-PCR and compared with those of the OVA-challenged TRPV1^(+/+)^ mice (Figure 2E). The OVA-challenged TRPV1^(−/−)^ mice exhibited decreased IL-4, IL-5, IL-6, and IL-17 expression and enhanced IFN-γ and IL-10 expression. Splenocyte culture profiles also showed decreased IL-4, IL-5, IL-6, and IL-17 levels (Figure 2F), which is a similar result to that obtained from the *in vitro* study of TRPV1^(−/−)^ CD4^+^ cells shown in Figure 1B. Since TRPV1 is expressed in various neuronal and non-neuronal cell types including immune cells, *in vivo* results may differ from those of *in vitro* T cell experiments. Nevertheless, our *in vivo* and *in vitro* studies confirmed that TRPV1 regulates the inflammatory response in OVA-challenged AR mice.

### TRPV1 antagonist suppresses the OVA-sensitized inflammatory response

**Figure 3:**
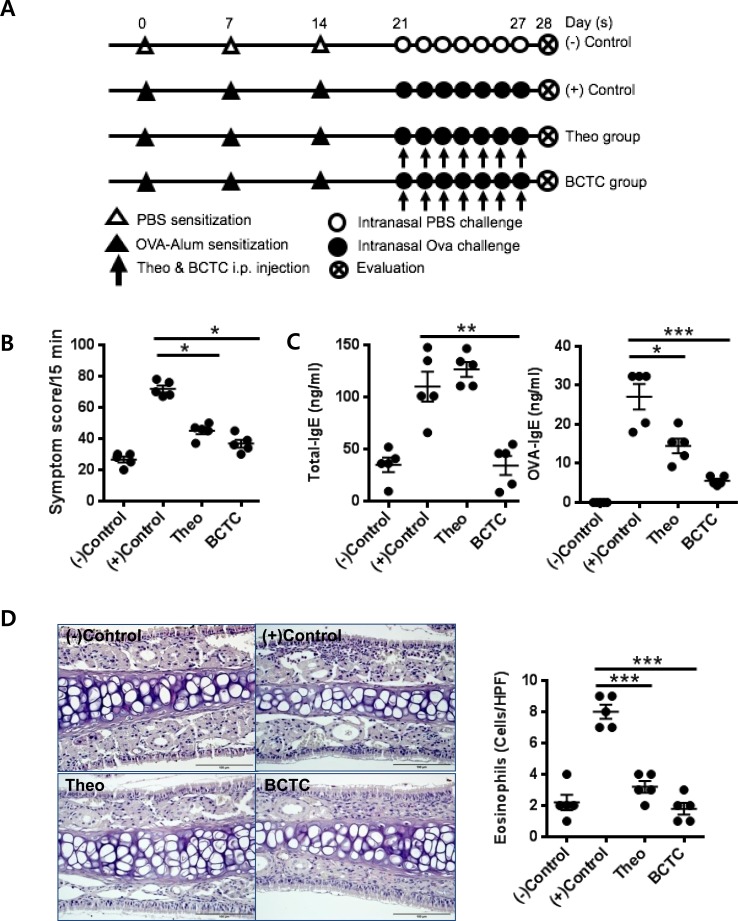

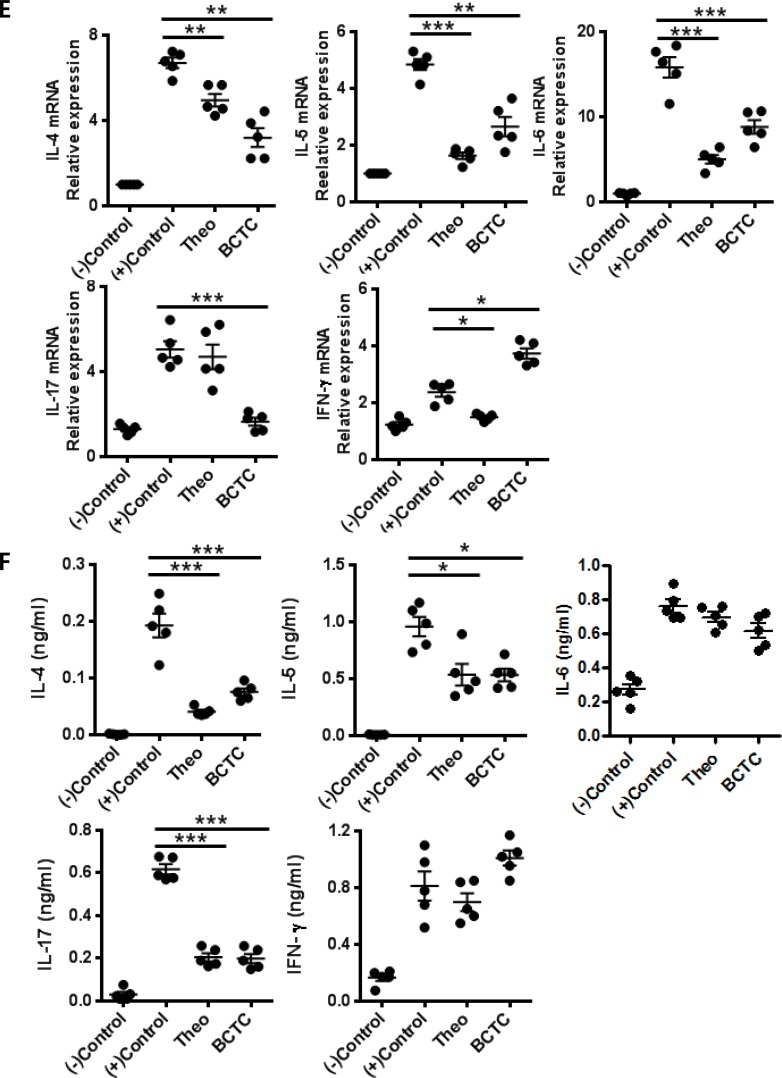
Effect of TRPV1 antagonist on the OVA-sensitized inflammatory response **A.** The experimental protocol of TRPV1 antagonist experiments. Theobromine and BCTC were used as TRPV1 antagonists **B.** Symptom scores of nasal rubbing and sneezing for 15 minutes after ovalbumin challenge **C.** Total and OVA-specific IgE levels in the serum as determined by ELISA, **D.** the infiltration of eosinophils, and the histopathological changes in the nasal mucosal septum as quantified by Sirius-red staining (400× magnification). **E.** mRNA expression of cytokines in the nasal mucosa as determined by the qRT-PCR using Taqman^®^ probe. **F.** The protein concentrations of cytokines as measured in the supernatants of splenocytes by ELISA. The spleens of the experimental mice were homogenized to obtain single-cell suspensions (3 × 10^6^ cells/ml) and cultured with or without OVA (1 mg/ml) for 72 h. The statistical *P* values are presented as * (*P* < 0.05), ** (*P* < 0.01), and *** (*P* < 0.001).

We further tested whether the TRPV1 antagonists BCTC and theobromine can suppress the allergic airway inflammatory response in OVA-challenged wild type mice (Figure 3A). The theobromine- and BCTC-treated OVA-challenged mice showed significantly reduced nasal rubbing and sneezing symptom scores as determined by monitoring the mice for a 15 min period, compared with those of the OVA-challenged mice without antagonist treatment (Figure 3B). Similarly, serum OVA-specific IgE levels (Figure 3C) and eosinophil infiltration in the nasal mucosa (Figure 3D) were significantly reduced in the OVA-challenged, TRPV1 antagonist-treated mice (*P* < 0.05, *P* < 0.001) compared with those of OVA-challenged mice without antagonist treatment. Cytokine levels in the nasal mucosa and spleen cell cultures were also evaluated. The OVA-challenged group without antagonist treatment had increased expression levels of the cytokines IL-4, IL-5, IL-6, IL-17, and IFN-γ in both the nasal mucosa and spleen cell cultures. We found that the TRPV1 antagonist treatments resulted in significant down-regulation of IL-4, IL-5, IL-6, and IL-17, altered expression of IFN-γ in the nasal mucosa (decreased in the theobromine group and increased in the BCTC group) (Figure 3E), and significant decreases in the expression of IL-4, IL-5, and IL-17 in spleen cell cultures (Figure 3F), relative to those of the OVA-challenged group without antagonist treatment. Although differences between mucosal and splenic cells were observed in the expression of cytokines, the results collectively indicated that the blockade of TRPV1 decreased the presence of Th2 and Th17 cytokines both locally and systemically in the OVA-challenged mouse model of AR.

### Clinical relevance of TRPV1-mediated inflammation in allergic rhinitis

**Figure 4:**
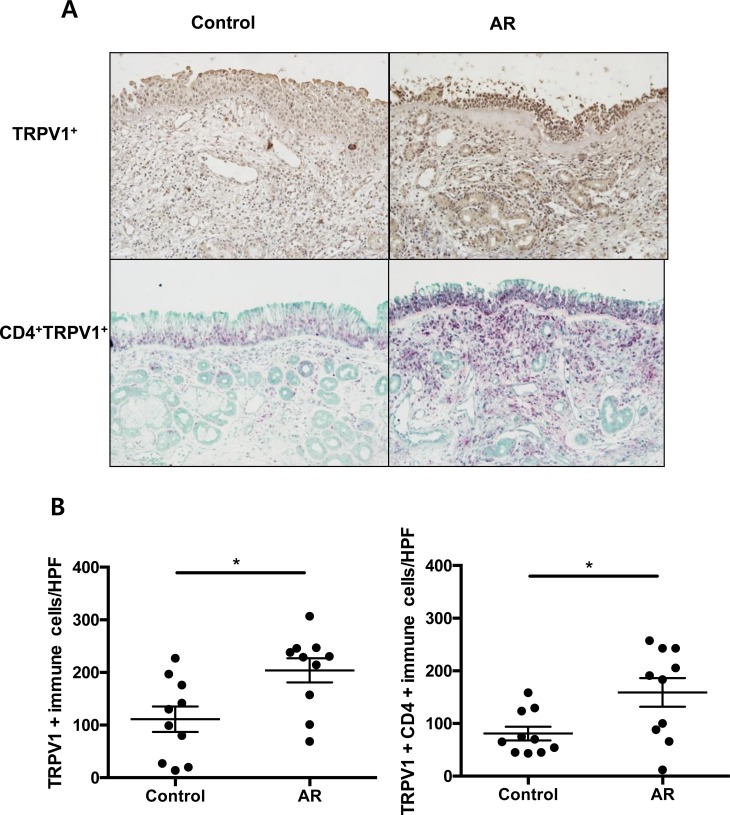
The TRPV1^+^ and TRPV1+CD4^+^ immune cells in patients with allergic rhinitis **A.** Immunohistochemical staining of TRPV1 (single staining) and CD4/TRPV1 (double staining) in patients with allergic rhinitis compared with control subjects **B.** Counts of TRPV1 + cells and TRPV1+ CD4+ cells in each groups. The statistical *P* values are presented as * (*P* < 0.05), and ** (*P* < 0.01).

To determine whether the expression of TRPV1 was altered in the inflammatory cells of the nasal turbinate mucosa from allergic rhinitis patients, immunohistochemistry was conducted in tissues from patients and normal controls. The number of TRPV1+ inflammatory cells was significantly higher in subjects with allergic rhinitis, compared with control subjects (Figure 4). In addition, we performed double immunohistochemistry staining of TRPV1 and CD4 and found that TRPV1 co-localized in CD4^+^T cells and that CD4^+^TRPV1^+^ inflammatory cells were more frequently observed in allergic rhinitis patients than in control subjects, suggesting the possibility that TRPV1 represents a therapeutic target in allergic rhinitis.

## DISCUSSION

TRPV1 is recognized as a Ca^2+^ permeable non-selective cationic channel functioning as a major neuronal heat receptor. TRPV1 is activated by low pH, endogenous lipids as well as many exogenous compounds including capsaicin, plant toxin (resiniferatoxin), mustard oil, and ethanol [14-17]. Allergen exposure also induces *de novo* TRPV1 expression in airway neurons [18]. Sensory C fibers expressing TRPV1 were reported to show high co-localization between the neurokinins calcitonin gene-related peptide and substance P [19]. TRPV1 signaling releases neuropeptides that can alter smooth muscle tone, increases airway secretions and submucosal edema, and activates inflammatory and immune cellular responses [20]. Additionally, TRPV1 on endothelial cells increases the trans-cellular migration and adhesion of leukocytes [21]. Taking these reports together, it is apparent that TRPV1 activation in the airway mucosa contributes to inflammation by inducing inflammatory mediators. However, whether TRPV1 signaling has a direct influence on T cell development or differentiation in allergic airway inflammation has not previously been elucidated.

Although some studies have previously examined the overexpression of TRPV1 in nasal airway inflammation [5], clinical trials have failed to demonstrate any clinical efficacy of the topical inhibition of TRPV1 [9-11]. It has been suggested that the reason underlying this lack of efficacy may be that neuronal TRPV1 was focused in human airway inflammation, rather than other cell types [6, 20, 22, 23]. Considering that immune cells may be one of the main targets for allergic inflammatory diseases and that TRPV1 expression and function have been recently reported in CD4^+^ T cells [24], we hypothesized that allergic airway inflammation might be regulated, in part, by the TRPV1 receptors on CD4^+^ T cells. In the present study, for the first time, we have shown that TRPV1 is expressed and functional on CD4^+^ T cells of the AR mouse model, and that the inhibition of TRPV1 expressed on CD4^+^ T cells may be a novel therapeutic target for allergic airway diseases. Interestingly, TRPV1 expression of inflammatory cells in the human turbinate mucosa was also up-regulated in AR patients compared with that observed in healthy controls. Therefore, further investigation regarding the role of TRPV1 on CD4^+^ T cells in airway allergic inflammation might offer another option for developing novel anti-inflammatory therapies.

To evaluate whether these immunologic changes were reproducible in an allergic situation *in vivo*, we utilized AR mice models. Both TRPV1^(−/−)^ and TRPV1 antagonist-treated mice exhibited decreased allergic inflammation and decreased Th2 cytokine production, showing that the inhibition of TRPV1 could reduce allergic inflammation. Nasal mucosal samples from these mice also showed decreased levels of Th2 and Th17 cytokine expression and increased expression of Th1 cytokines. These results are consistent with previous studies that investigated the effects of TRPV1 inhibition in a mouse model of asthma [25-27]. Rehman *et al*. (2013) reported that the knockdown of TRPV1 using siRNA attenuated allergic inflammation in their IL-13 driven asthma model [25] and Delescluse *et al*. (2012) also showed that treatment with a TRPV1 antagonist reduced histamine-induced airway hyper-responsiveness in OVA-sensitized guinea pigs [26]. Another study using an IL-31-induced atopic dermatitis model also showed decreases in inflammation and itching in TRPV1 knockout mice [27]. One interesting study demonstrated the existence of a close relationship between TRPV1 and TSLP. TSLP is a Th2 response-driving innate cytokine released by epithelial cells, and the authors showed that the knockdown of TRPV1 decreased the release of TSLP in airway epithelial cells. This TRPV1/TSLP pathway could explain the occurrence of decreased Th2 inflammation following TRPV1 inhibition. On the other hand, we observed IFN-γ gene expression was modestly up-regulated in the nasal tissues of TRPV1-inhibited AR mice models (Figures 2E and 3E). One possible explanation, though mechanism was not elucidated in this study, is that the decrease of IL-4 as a negative regulator of IFN-γ reduces the activation of GATA-3, which may facilitate IFN-γ production by allergen stimuli as a counteract.

In conclusion, we found *in vitro* and *in vivo* evidence indicating that TRPV1 increases allergic inflammation in nasal mucosa which is also supported by previous studies. Obtaining an increased understanding of the role of TRPV1 in patients with AR might offer another option for the development of novel anti-inflammatory therapies for allergic diseases.

## MATERIALS AND METHODS

### Animals

The experiments were performed in six- to eight-week-old female BALB/C and C57BL/6 (TRPV1^(+/+)^) mice obtained from Korea Biolink Co. (Eumsung, Korea). TRPV1 knockout mice (B6.129X1-*Trpv1^tm1Jul^*/J: TRPV1^(−/−)^) were purchased from the Jackson Laboratory (Bar Harbor, Maine, USA). These animals were maintained in a specific pathogen-free biohazard containment facility. All of the animal experiments were conducted following the guidelines and ethics of Institutional Animal Care at the Clinical Research Institute (DKU-13-114).

### Sensitization and challenge

The OVA sensitization and challenge procedure was based on a previously established allergic rhinitis model [28]. In addition, selected groups of mice were treated with an intraperitoneal injection of the TRPV1 antagonist theobromine (0.64 mg/day for one week) and BCTC (0.06 mg/day for one week) 3 h before the intranasal OVA challenge.

### Symptom score and tissue preparation

After the final OVA challenge on day 27, a blind observer recorded the frequencies of sneezing and nasal rubbing for 15 minutes. The mice were then sacrificed after the last OVA challenge. After perfusion with 4% paraformaldehyde, the heads of five mice from each group were removed en bloc and then fixed in 4% paraformaldehyde. After removing the nasal cavity from the head of the other five mice, the nasal mucosa was meticulously removed using a small curette. The nasal mucosa was immediately immersed in liquid nitrogen or stored at −70°C until use for quantitative real time-polymerase chain reaction (qRT-PCR).

### Assessment of eosinophil infiltration

For evaluation of the nasal histology, the nasal tissues fixed in 4% paraformaldehyde were decalcified, embedded in a paraffin block and sectioned coronally into 5-μm-thick sections approximately 5 mm from the nasal vestibule. Each section was stained with Sirius red or hematoxylin and eosin, the number of eosinophils on both sides of the mucosa was counted. The number of eosinophils in the submucosal area of the whole nasal septum was counted under a light microscope with 400× magnification.

### Purification of naïve CD4^+^ T lymphocytes from mouse spleen

CD4^+^ T lymphocytes were isolated from the spleen of BALB/C, C57BL/6 (TRPV1^(+/+)^ and TRPV1^(−/−)^) mice using a MACS separator magnetic column with restrained mouse anti-CD4-labeled cells. The isolated CD4^+^ T cells were pre-incubated with different concentrations of BCTC (0.1, 1 and 10 μg/ml) for 1 h and then co-stimulated with or without αCD3/CD28-pre-coated well plates. After 72 h of stimulation with the antibodies, the CD4^+^ T cell supernatant was collected and stored at −70°C until assessment of the cytokine levels. Jurkat T cells were cultured in RPMI-1640 media supplemented with 10% FBS, 2 mM L-glutamine, 100 U/mL penicillin and 100 μg/mL streptomycin solutions. Jurkat T cells were treated with or without αCD3/CD28-pre-coated antibodies for different times (15, 30 and 60 min) after 1 h of pre-incubation with different concentrations of BCTC (0.1, 1 and 10 μg/mL).

### Estimation of systemic cytokine profiles

The spleen of experimental mice prepared with single-cell suspensions was plated into 24-well cell culture plates at a final concentration of 3×10^6^ cells/mL in RPMI 1640 media (Gibco, Grand Island, NY, USA). The cells were incubated in a CO_2_ incubator at 37°C and stimulated with OVA (1 mg/mL) for 72 h. The culture supernatant was collected and stored at −70°C until assessment of the cytokine levels. Similarly, the supernatants of CD4^+^ T cells stimulated *in vitro* with αCD3/CD28 for 72 h were collected. The concentrations of IL-4, IL-5, IL-6, IL-10, IL-17 and IFN-γ in the supernatants were determined using ELISA kits (BD Technologies).

### Estimation of serum total immunoglobulin (Ig)E and OVA-specific IgE, IgG1, and IgG2a

The serum levels of total and OVA-specific IgE were measured by solid-phase ELISA. The detailed procedure was described in our previous research manuscript [28]. Analysis of the OVA-specific IgG1 and IgG2a was performed using Nunc 96-well immunoplates coated with 100 μg/mL OVA in coating buffer (carbonate-bicarbonate) through overnight incubation at 4°C. To detect the OVA-specific IgG1 and IgG2a levels, serially diluted serum samples were incubated with biotinylated rat anti-mouse IgG1 (BD Pharmingen, San Jose, CA, USA) and IgG2a (BD Pharmingen), respectively, prior to the addition of streptavidin-HRP. The SureBlue TMB Micro-well Substrate (KPL, Gaithersburg, MD, USA) was used for peroxidase detection, and the color was allowed to develop for 30 min. After the reaction was stopped by the addition of 1 M HCL, the OD was measured using a microplate reader (Molecular Devices, Silicon Valley, CA, USA). The endpoint titer of OVA-specific IgGs is expressed as the reciprocal of the log^2^ titer.

### Quantitative real-time PCR analysis of the systemic cytokine profiles

The total RNA was extracted using the TRIzol reagent (Invitrogen) according to the manufacturer's instructions. Equivalent amounts of RNA were reversed-transcribed using the iScript cDNA Synthesis Kit (Bio-red Laboratories; Hercules, CA, USA). The mRNA expression analysis was performed using an Applied Biosystem 7500 Real-Time PCR System (Applied Biosystems, Foster City, CA, USA). For analysis of IL-4 (Mm00445258_g1), IL-5 (Mm01290072_g1), IL-6 (Mm00446190­_m1), IL-10 (Mm00439616_m1), IL-17 (Mm00439618_m1), IFN-γ (Mm99999071_m1), and GAPDH (Mm03302249_g1), PDAR (pre-developed assay reagent) kits of the corresponding primers and probes were purchased from Applied Biosystems (Foster City, CA, USA). The relative gene expression was calculated using the 2^−ΔΔCt^ method.

### Quantification of immunoblotting

A total of 5×10^6^ CD4^+^ T cells in each group were extracted from the cytosolic and nuclear fractions using cell lysis buffer. The supernatants were separated, and the protein concentration in the fractions was quantified using a Bradford quantitative protein assay kit (Applygen Technologies Inc., China). The total protein lysates (40 μg per each well) were loaded and subjected to 10-12% SDS-PAGE before being electro-transferred to polyvinylidene difluoride membranes (PVDF) at 100 V for 1 h. The membrane was blocked with 5% nonfat milk in Tris-buffered saline supplemented with 0.05% Tween 20 (pH 7.6) at 4°C for 2 h and incubated with anti-mouse and anti-human polyclonal antibodies, including antibodies against ERK, phospho-ERK, JNK, phospho-JNK, NFAT1, p65 (NF-κB), phospho-p65 (NF-κB), p38, phospho-p38 and β-actin (1:1,000, Santa Cruz Biotechnology, Santa Cruz, CA, USA and Cell Signaling, USA), overnight at 4°C. The membrane was incubated with horseradish peroxidase-conjugated anti-mouse and anti-rabbit secondary antibody (1:2,000, Santa Cruz Biotechnology, Santa Cruz, CA, USA) at room temperature for 2 h. After extensive washing, the blot was developed using an ECL chemiluminescent detection kit (TransGen Biotech Co. Ltd., China) according to the manufacturer's instructions. The X-ray films were scanned and quantified by Gel-Doc (Bio-red) using image analysis software.

### FACS analysis

Cells obtained from the spleen and cervical lymph node were stained for anti-CD4-FITC (eBioscience, San Diego, CA, USA) anti-TRPV1 (Alomone Labs, Jerusalem, Israel), biotinylated Goat Anti-Rabbit IgG (Vector Laboratories, Burlingame, CA, USA) and Streptavidin−Cy3 (Sigma-Aldrich, St. Louis, MO, USA). The stained cells were analyzed using a Accuri flow cytometer (BD Biosciences - Immunocytometry Systems, San Jose, California, USA).

### Confocal laser scanning microscopy analysis

CD4^+^ T cells (3 × 10^6^) were fixed and permeabilized using CytoFix/CytoPerm Plus (BD Biosciences, Milano, Italy) for 30 min at 4°C. The cells were washed twice and incubated with rabbit anti-TRPV1 and mouse anti-CD4 monoclonal antibodies (1:50) for 1 h at 4°C. After washing twice, the cells were incubated with FITC-conjugated anti-rabbit IgG and Cy3-conjugated anti-mouse IgG polyclonal antibodies (1:100) for 1 h at 4°C, and washed twice. The nuclei were counterstained with DAPI, and the samples were mounted on slides and analyzed with an LSM510 META confocal laser scanning microscope (Zeiss, Broadway, Baltimore, MD, USA). The fluorochrome was excited with the 600 line of an argon-krypton laser and imaged using a 488(FITC) and 564(Cy3)-nm bandpass filter. Serial optical sections were prepared at 1-mm intervals through the cells.

### Human study

#### Subjects

The sinonasal tissues, turbinate mucosa were obtained through septoplasty from patients with allergic rhinitis (AR; n=10) and normal control (control; n=10) subjects. All patients provided written informed consent. This study was approved by the internal review board of Dankook University Hospital. Exclusion criteria were as follows: (1) younger than 18 years of age, (2) prior treatment with antibiotics, systemic or topical corticosteroids, or other immune-modulating drugs for 4 weeks before surgery, (3) combined with other nasal diseases including rhinosinusitis, antrochoanal polyp, allergic fungal sinusitis, cystic fibrosis, or immotile ciliary disease. Allergic rhinitis and non-allergic rhinitis were defined as a minimum of 2 nasal complaints (itching, nasal obstruction, rhinorrhea, and sneezing) for more than 1 hour a day and for more than 1 year, with positive skin prick test response and with negative SPT responses, respectively. Control tissues were obtained from patients without any nasal inflammatory diseases with negative SPT responses during rhinologic surgeries. Patient characteristics are summarized in Table 1.

**Table 1:** Patient characteristics

	Control	Allergic rhinitis
Total no. of subjects	N = 10	N = 10
Age (yr), mean (SEM)	37.0 (20-58)	23.8 (18-59)
Skin prick test positivity (N)	0	10
Asthma (N)	0	0
Nasal symptoms, mean (SEM)		
Nasal congestion, mean	0	3.4 ±1.0
Rhinorrhea, mean	0	3.1 ±1.3
Itching, mean	0	3.2 ±1.2
Sneezing, mean	0	3.2 ±1.4

#### Immunohistochemistry of TRPV1 expression

Nasal mucosal sections and human sinonasal tissues were immunostained for TRPV1 using avidin-biotinylated-horseradish peroxidase (HRP) complex kits (Vector Laboratories, Burlingame, CA, USA). After deparaffinization in xylene, the sections were rehydrated with different gradients of ethanol. After washing in PBS, the sections were blocked with 1% normal goat serum and then treated with primary rabbit polyclonal antibody for TRPV1 with both human and mouse reactivity (1:50, Cat # NBP1-71774; Novus Biologicals, USA) overnight at 4°C in a humidified chamber. After washing in PBS, the sections were incubated for 60 min at room temperature with secondary antibody (biotin-conjugated goat anti-rabbit IgG, 1:100 dilutions). The sections were then incubated with avidin-biotinylated-HRP complex for 30 min at room temperature, rinsed in PBS, and then developed with 0.027% 3,3-diaminobenzidine tetrahydrochloride (Sigma, Inc.) and 0.003% hydrogen peroxide. The sections were then counterstained with hematoxylin (Sigma, Inc.), and the images were captured with a brightfield microscope at 400× magnification. The Image J software (U.S. National Institutes of Health, Bethesda, MD, USA) was used to quantify the strength of the immunohistochemical staining in each section.

To confirm co-localization of CD4 and TRPV1, sequential IHC was employed using polymer-HRP and alkaline phosphatase (AP) kits to detect mouse and rabbit primary antibodies for human tissue with permanent-Red and Emerald (Polink DS-MR-Hu C2 Kit; Golden Bridge International Labs). Primary antibodies against cellular phenotypic markers included mouse anti-CD4 (1:50; Santa Cruz Biotechnology) and rabbit anti-human TRPV1 (1:50, Cat # NBP1-71774; Novus Biologicals, USA). These antibodies were mixed each other, applied to the tissue, and then incubated for 30-60 min. Polymer mixtures were made by adding AP polymer anti-mouse IgG and polymer-HRP anti-rabbit IgG at a 1:1 ratio and applied to cover each section. Unless noted otherwise, all manufacturer's instruction was followed. Finally, slides were counterstained with hematoxylin. The numbers of positive cells in the submucosa were counted in the five densest visual fields (X 400) by two independent observers, and the average values were determined.

### Statistical analysis

The statistical analyses were performed using GraphPad Prism 6 software tools. The results were analyzed using Newman-Keuls test, and all pairs of columns were compared with one-way analysis of variance (ANOVA). The values of the comparisons between groups, including negative and positive groups, are expressed as the means ± SEM. The *P* values obtained through one-way ANOVA and Newman-Keuls test that were less than 0.05, 0.01 and 0.001 are represented by *, **, *** to indicate statistical significance.

## SUPPLEMENTARY MATERIAL FIGURE


