# Supplementary material for: The role of TRPV1 in the CD4+ T cell-mediated inflammatory response of allergic rhinitis
Source: Oncotarget. 2015 Dec 18;7(1):148–60. doi: 10.18632/oncotarget.6653 (PMC4807989; doi:10.18632/oncotarget.6653)
Supplement: Supplementary file 1 [file oncotarget-07-0148-s001.pdf]

The Role of TRPV1 in the CD4$^+$ T cell-mediated inflammatory response of allergic rhinitis

Supplementary Material

Methods

Quantitative real time RT-PCR by TRPV1 expression

The total RNA from the tissue samples was extracted using the TRIzol reagent (Invitrogen) according to the manufacturer's instructions. Equivalent amounts of RNA (1 µg) from the total RNA were reversed-transcribed to cDNA using the iScript cDNA Synthesis Kit (Bio-red Laboratories; Hercules, CA, USA). The mRNA expression analysis was performed using a Applied Biosystem 7500 Real-time PCR System (Applied Biosystems, Foster City, CA, USA) with the AccuPower 2X Greenstar qPCR Master kit (Bioneer Corporation, Daejeon, Republic of Korea). The quantitative real-time PCR assay was based on appropriate primers that were specifically designed for mice and humans. The primers, which were purchased from Bioneer Corporation (Daejeon, Republic of Korea) were as follows: mouse TRPV1 forward, 5'-CGAGGATGGGAAGAATAACTCACTG-3'; and reverse, 5'-GGATGATGAAGACAGCCTTGAAGTC-3'; human TRPV1 forward, 5'-AGCCACCTCAAGGAGTATGTG-3'; and reverse, 5'-CCCGAACAAGAAGACGATG-3'; and housekeeping gene (GAPDH) forward, 5'-CCTCGTCCCGTAGACAAAATG-3'; and reverse, 5'-TCTCCACTTTGCCACCTGCAA-3'. The cycling conditions were as follows: stage 1, 50°C for 2 min; stage 2, 95°C for 10 min; and stage 3, 40 cycles of 95°C for 15 s and 60°C for 1 min. The gene expression levels were normalized to the housekeeping gene GAPDH and were treated using the $2^{-\Delta\Delta Ct}$ method.

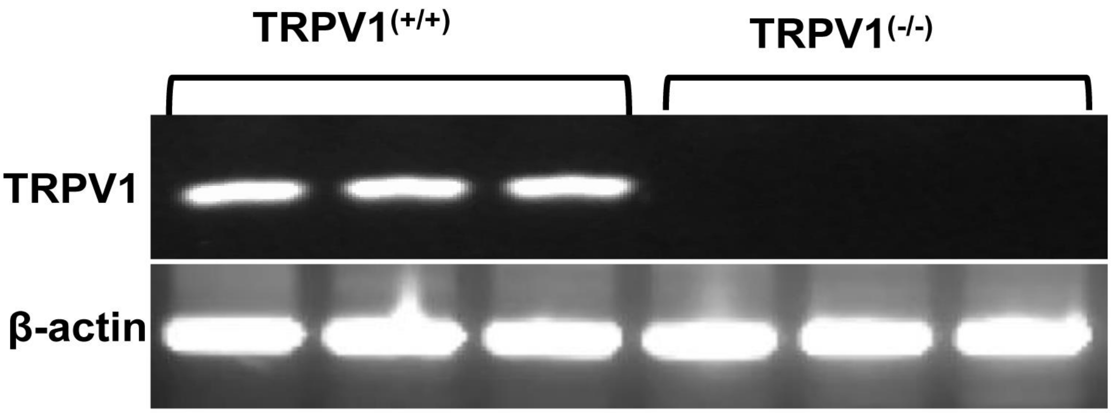

Supplementary Figure 1. The mRNA expressions of TRPV1 of the experimental mouse confirmed by conventional PCR. The samples were collected from ear skin of TRPV1$^{(+/+)}$ and TRPV1$^{(-/-)}$ mice.
